# Ameloblastomas mimicking apical periodontitis: a case series

**DOI:** 10.4317/medoral.25338

**Published:** 2022-06-05

**Authors:** Giuliana Soimu, Juliana de Noronha Santos Netto, Águida Maria Menezes Aguiar Miranda, Danyel Elias da Cruz Perez, Luciana Armada, Fábio Ramoa Pires

**Affiliations:** 1 DDS, MSc, Post-graduation Program in Dentistry, Estácio de Sá University, Rio de Janeiro/RJ, Brazil; 2 DDS, PhD, Professor, Stomatology, Universidade Federal do Rio de Janeiro, Rio de Janeiro/RJ, Brazil; 3 DDS, MSc, Staff, Stomatology, Brazilian Dental Association, Rio de Janeiro/RJ, Brazil; 4 DDS, PhD, Professor, Oral Pathology, School of Dentistry, Universidade Federal de Pernambuco, Recife/PE, Brazil; 5 DDS, PhD, Professor, Post-graduation Program in Dentistry, Estácio de Sá University, Rio de Janeiro/RJ, Brazil; 6 DDS, PhD, Professor, Oral Pathology, Dental School, Rio de Janeiro State University and Professor, Post-graduation Program in Dentistry, Estácio de Sá University; Rio de Janeiro/RJ, Brazil

## Abstract

**Background:**

Ameloblastomas are benign odontogenic tumors that can eventually mimic the clinical and radiological features of apical periodontitis. The aim of the present study was to evaluate the clinical, radiological and histological characteristics from a series of ameloblastomas mimicking apical periodontitis diagnosed in a 14-year period.

**Material and Methods:**

all cases histologically diagnosed as ameloblastomas from 2005 to 2018 presenting a clinical diagnosis of periapical lesion of endodontic origin were selected for the study. Clinical, radiological and histological characteristics from all cases were tabulated and descriptively and comparatively analyzed.

**Results:**

Twenty cases composed the final sample, including 18 solid and 2 unicystic ameloblastomas. Mean age of the affected patients was in the fifth decade with predilection for males (72%). The most common anatomical location was the posterior mandible (55%) and most cases presented a radiolucent unilocular (80%) well-defined (95%) image. Most cases were asymptomatic, but the presence of local swelling and bone cortical rupture were common.

**Conclusions:**

Ameloblastomas mimicking periapical lesions of endodontic origin are mostly diagnosed in adult males as well-defined radiolucent unilocular lesions producing local swelling and bone cortical rupture.

** Key words:**Ameloblastoma, apical periodontitis, cyst, differencial diagnosis, granuloma, periapical lesion.

## Introduction

Most lesions observed in the periapical region are associated with inflammatory processes derived from the dental pulp and its consequent necrosis, as a result of caries or dental trauma. However, there is a wide variety of conditions located in the apical region that can simulate inflammatory periapical diseases. These conditions, known collectively as non endodontic periapical lesions (NEPL), can represent 2 to 27% of the injuries associated with the periapical area submitted to histological analysis (1-7).

Ameloblastomas are locally agressive epithelial odontogenic tumors characterized by uni or multilocular radiolucent images with predilection for the posterior mandible and can be located in close relationship to the adjacent teeth roots (8-10). Due to their local aggressive behavior early and prompt diagnosis is essential, avoiding misinterpretation as inflammatory conditions leading to unnecessary treatments and delay in establishing correct diagnosis and appropriate surgical treatment (11,12).

Although this is an essential subject for the endodontist background, only few case reports and very few small case series have been published focusing on ameloblastomas mimicking apical periodontitis (AMAP) (12-19). Thus, the aim of the present study is to report a series of AMAP with a detailed clinical, radiological and histological analysis.

## Material and Methods

This is a cross-sectional, retrospective and descriptive study that was conducted in accordande with the Declaration of Helsinki and was approved by the Ethics in Research Committee (protocol 2.223.941). All specimens diagnosed as ameloblastomas in the Oral Pathology laboratory, Dental School, Rio de Janeiro State University, from 2005 to 2018 were reviewed. All cases including a clinical diagnosis compatible with apical periodontitis (such as inflammatory periapical lesion, periapical granuloma, periapical or radicular cyst, and dentoalveolar abscess), provided by the responsible clinician and/or oral and maxillofacial surgeon based on panoramic radiographs, were selected for further analysis.

Laboratory records, including data derived from biopsy submission forms, were reviewed and information on age (in years), gender, location of the tumor (maxilla or mandible; anterior or posterior region), symptoms, local swelling, and radiological features (pattern and size of the image, and relationship with the roots of the adjacent teeth by using panoramic radiographs) were retrieved. Hematoxylin and eosin stained histological sections from all cases were reviewed for diagnosis confirmation and for further histological analysis. Cases presenting few or no clinical and radiological informations, and cases in which the histological findings were insufficient for the conclusive diagnosis of ameloblastoma were excluded from the final sample.

Data obtained from the clinical, radiological and histological analysis were tabulated and descriptively and comparatively analyzed through the use of the Statistical Program for Social Sciences software (SPSS, IBM, version 20). Statistical significance was established at 5% (*p*<0.05).

## Results

In the 14-year period (from 2005 to 2018), 20 AMAP were retrieved from the laboratory files, including 18 solid ameloblastomas and two unicystic ameloblastomas. Solid ameloblastomas affected 13 males (72%) and 5 females (28%). The two unicystic ameloblastomas affected males. Mean age of patients with solid ameloblastomas was 41.4 years (standard deviation - SD - 12.77, ranging from 17 to 59 years), and both unicystic ameloblastomas were diagnosed in adults (Table 1). Mean age of the affected males (43.8 years) was higher than affected females (35.4 years) (*p*=0.223).

Fourteen solid ameloblastomas (82%) and the two unicystic ameloblastomas were associated with local swelling. The mean radiological size for solid and unicystic ameloblastomas was 37.1 mm and 40 mm, respectively. Mean radiological size of the tumors was larger in females (53.8 mm) than in males (32 mm) (*p*=0.107). Symptoms were reported in 3 (20%) solid ameloblastomas, and included pain, suppuration and paresthesia. Mobility and/or displacement of the associated adjacent teeth were identified in 7 (47%) solid ameloblastomas and in both unicystic ameloblastomas. Solid ameloblastomas were located in the posterior mandible (10 cases, 55%), anterior mandible (3 cases, 17%), posterior maxilla (3 cases, 17%), and anterior maxilla (2 cases, 11%); each unicystic ameloblastoma affected the posterior and anterior mandible (Table 1). Mean time of onset in solid ameloblastomas was 24 months (SD - 23.63, ranging from 5 to 60 months); both patients affected by unicystic ameloblastomas were not aware about the precise time of onset.

**Figure 1:**
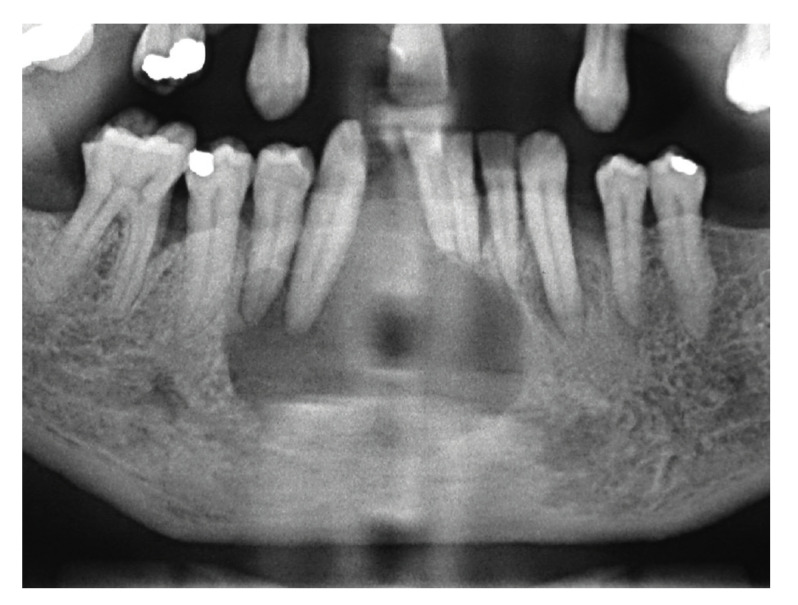
Solid ameloblastoma mimicking apical periodontitis. Well-defined unilocular radiolucency in the anterior mandible extending from the left lateral incisor to the right first premolar, causing displacement of lower incisors and right canine.

**Figure 2:**
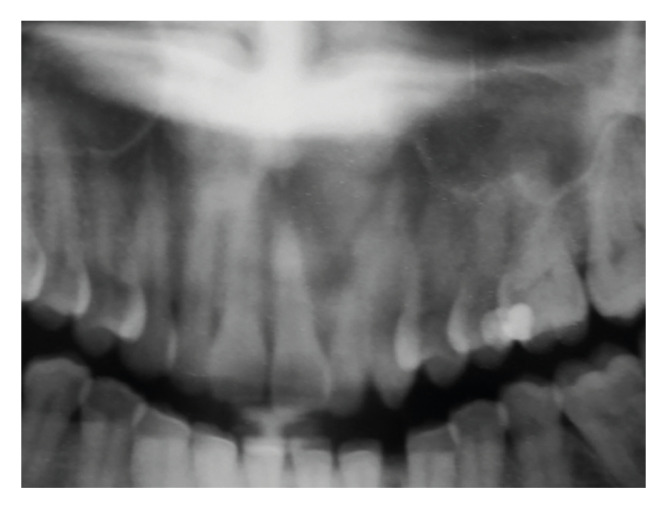
Solid ameloblastoma mimicking apical periodontitis. Well-defined unilocular radiolucency in the anterior maxilla extending from the right central incisor to the left first premolar, casuing displacement of the upper left central and lateral incisors (Courtesy: Drs. Marília Heffer Cantisano, Geraldo Oliveira Silva-Júnior, and Thays Teixeira - Stomatology section, Policlínica Piquet Carneiro, Rio de Janeiro State University, Rio de Janeiro, Brazil).

**Figure 3:**
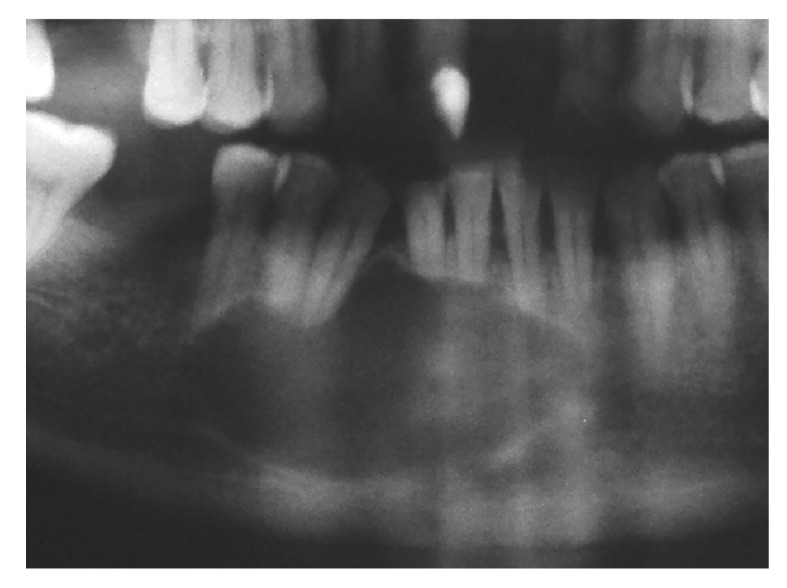
Solid ameloblastoma mimicking apical periodontitis. Well-defined unilocular radiolucency in the anterior mandible extending from the left lateral incisor to the right second premolar, causing teeth displacement and root resorption (Courtesy: Drs. Roberto Bastos and Henrique Martins da Silveira - Oral and Maxillofacial Surgery, Hospital Pedro Ernesto, Rio de Janeiro State University, Rio de Janeiro, Brazil).

Solid and unicystic ameloblastomas presented as unilocular radiolucencies in 78% and 100% of the cases, respectively (Fig. 1, Fig. 2 and Fig. 3). Cortical bone rupture was observed in 14 solid (82%) and in one (50%) unicystic ameloblastoma. The radiological limits were considered well-defined in 16 solid (94%) and in both unicystic ameloblastomas. Endodontic status of the associated teeth was evaluated in 14 solid ameloblastomas and showed that the teeth had endodontic treatment in only two cases (14%). Clinical differential diagnosis included periapical cyst/granuloma (20 cases), odontogenic keratocyst (7), unicystic ameloblastoma (6), residual cyst (5), and nasolabial cyst, dentigerous cyst, lateral periodontal cyst, aneurismal bone cyst, adenomatoid odontogenic tumor, central giant cell lesion and hemangioma (1 case each).

**Table 1:**
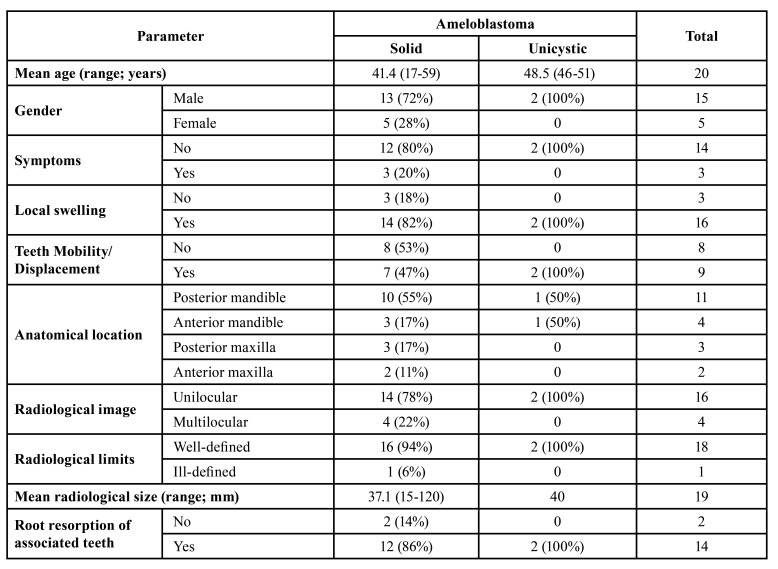
Distribution of the clinical and radiological information according with the type of ameloblastoma.

The predominant histological pattern for solid ameloblastomas was the follicular subtype (12 cases, 67%), followed by plexiform (3 cases, 17%), acanthomatous (2 cases, 11%), and adenoid subtypes (1 case, 5%). Both unicystic ameloblastomas were classified as luminal. No statistically significant differences were found when comparing the location of the tumors and their histological pattern with gender of the affected patients.

## Discussion

NEPL represent up to 27% of the conditions located in the periapical region submitted to histological analysis (Table 2). In this group, odontogenic keratocysts, ameloblastomas, nasopalatine cysts, dentigerous cysts, glandular odontogenic cysts, fibro-osseous lesions and giant cell lesions are the most common reported diseases. Ameloblastomas represent 1.2% to 11.5% of all NEPL according with the most recently published literature (1-7).

**Table 2:**
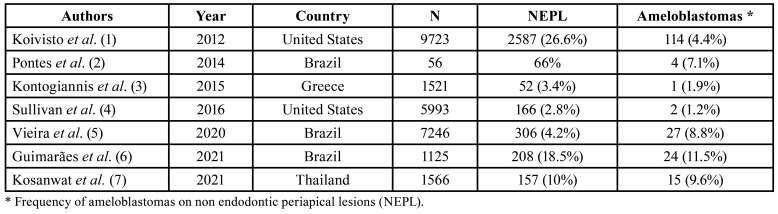
Frequency of non endodontic periapical lesions (NEPL) reported in the literature, with emphasis on ameloblastomas.

The present study focused on a descriptive and comparative analysis of the clinical, radiological, and histological characteristics of AMAP comparing the results with those described for ameloblastomas in general (8-10,20). Few studies have been directed to the analysis of periapical and periradicular ameloblastomas and their similarities with apical periodontitis (15,21). Most papers included isolated AMAP case reports (12,22), and there was only one previously published short series (17). The present study seems to be the largest series of AMAP including both solid and unicystic ameloblastomas published up to now.

Solid AMAP included in the present study affected mostly adult males, similarly to solid ameloblastomas in general (8,10,15,20). Mean age of unicystic AMAP in the present study, however, was higher than the mean age of usual unicystic ameloblastomas (8,10). Solid ameloblastomas were the predominant type and the follicular was the most common histological pattern, followed by the plexiform subtype, in agreement with most studies (10,15,20). In contrast, Fregnani *et al*. (9) reported that the plexiform subtype represented 53% of their sample.

AMAP were preferentially located in the posterior mandible, as it has been shown for ameloblastomas in general (8-10,15,20). It is worth mentioning, however, that 5 cases (25%) affected the maxilla, reinforcing that ameloblastomas should be considered in the differential diagnosis of endodontic lesions also in this anatomical location. Most ameloblastomas were associated with local swelling but few were symptomatic, similarly to usual ameloblastomas (8-10,21).

The mean radiological size of the AMAP included in the present study was lower than the mean average size of ameloblastomas (8,9). Most cases showed a unilocular radiolucent image, contrarily to most ameloblastomas (9,10,20). This difference could be atribuTable to the applied sample selection, focusing on ameloblastomas that had a presumptive clinical diagnosis of apical periodontitis. Most radiological limits were considered well-defined, similarly to what has been demonstrated for ameloblastomas in general (8,10). Even being characterized by relatively small well-defined unilocular images, most AMAP included in the present study showed cortical bone disruption, reinforcing the local aggressiveness of these tumors. In the present study, tooth displacement and/or mobility and root resorption were frequently found in both solid and unicystic ameloblastomas. Ide *et al*. (15) reported the presence of root resorption in 5 of the 14 ameloblastomas included in their study.

The present study highlights the importance of the correct diagnosis of incipient periapical lesions, whether endodontic or not, correlating the clinical and radiological findings, and when indicated, with the histological features of the surgical specimen (2). In addition to ameloblastomas, the other presumptive clinical diagnosis suggested for the cases included in the present sample were similar to those reported in the literature (16,19). Curiously, solid ameloblastoma was not provided as a clinical differential diagnosis in none of the present reported cases. It is essential for clinicians, particularly endodontists, to be aware of the possible differential diagnosis of periapical radiolucent lesions, considering their clinical and radiological characteristics. Although intraosseous lesions that are not related to pulpal conditions represent a small percentage of periapical lesions, an accurate diagnosis is essential for the correct treatment protocol, taking into account their individual biological behavior, as in the case of ameloblastomas (4).

Clinicians should be aware of the main clinical and radiological features that would suggest the possibility of AMAP and alert for its inclusion in the differential diagnosis of periapical radiolucencies. The present results have shown that AMAP were more common in males in their fifties, with most cases located in the posterior mandible, although 25% of the cases affected the maxilla. They were mostly solid ameloblastomas presenting as an asymptomatic swelling associated with teeth mobility. Most cases were characterized by well-defined unilocular radiolucencies, presenting a mean size of 40 mm, and associated with tooth displacement, root resorption, and cortical bone rupture.
